# 2-(4-Chloro-1*H*-indol-3-yl)acetonitrile

**DOI:** 10.1107/S1600536811054079

**Published:** 2011-12-21

**Authors:** Mei-Ling Pan, Xiang Li, Yang-Hui Luo

**Affiliations:** a College of Chemistry and Chemical Engineering, Southeast University, Nanjing 210096, People’s Republic of China

## Abstract

The title compound, C_10_H_7_ClN_2_, contains two approximately planar mol­ecules, *A* and *B* (r.m.s. deviations = 0.039 and 0.064 Å, respectively) in the asymmetric unit. In the crystal, N—H⋯N hydrogen bonds link the mol­ecules into *C*(7) chains of alternating *A* and *B* mol­ecules propagating along the *a*-axis direction. The crystal used for the data collection was found to be a racemic twin.

## Related literature

For a related structure, see: Ge *et al.* (2012 ▶).
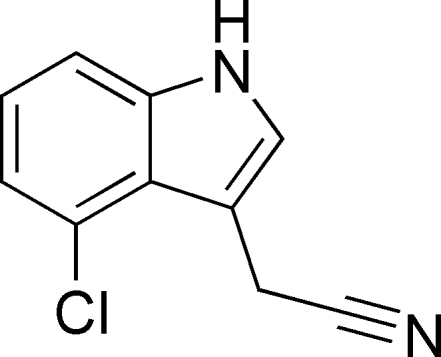


## Experimental

### 

#### Crystal data

C_10_H_7_ClN_2_
*M* *_r_* = 190.63
Orthorhombic, 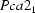

*a* = 7.5091 (15) Å
*b* = 11.041 (2) Å
*c* = 21.380 (4) Å
*V* = 1772.6 (6) Å^3^
*Z* = 8
Mo *K*α radiation
μ = 0.38 mm^−1^
*T* = 293 K
0.33 × 0.25 × 0.20 mm

#### Data collection

Rigaku SCXmini diffractometer
Absorption correction: multi-scan (*CrystalClear*; Rigaku, 2005 ▶) *T*_min_ = 0.893, *T*_max_ = 0.927
17075 measured reflections
4057 independent reflections
2753 reflections with *I* > 2σ(*I*)
*R*_int_ = 0.058
2 standard reflections every 150 reflections  intensity decay: none

#### Refinement

*R*[*F* ^2^ > 2σ(*F* ^2^)] = 0.048
*wR*(*F* ^2^) = 0.111
*S* = 1.03
4057 reflections
236 parameters
1 restraint
H-atom parameters constrained
Δρ_max_ = 0.27 e Å^−3^
Δρ_min_ = −0.22 e Å^−3^
Absolute structure: Flack (1983 ▶), 1968 Friedel pairs
Flack parameter: 0.66 (10)

### 

Data collection: *CrystalClear* (Rigaku, 2005 ▶); cell refinement: *CrystalClear*; data reduction: *CrystalClear*; program(s) used to solve structure: *SHELXS97* (Sheldrick, 2008 ▶); program(s) used to refine structure: *SHELXL97* (Sheldrick, 2008 ▶); molecular graphics: *DIAMOND* (Brandenburg & Putz, 2005 ▶); software used to prepare material for publication: *SHELXL97*.

## Supplementary Material

Crystal structure: contains datablock(s) I, global. DOI: 10.1107/S1600536811054079/hb6554sup1.cif

Structure factors: contains datablock(s) I. DOI: 10.1107/S1600536811054079/hb6554Isup2.hkl

Supplementary material file. DOI: 10.1107/S1600536811054079/hb6554Isup3.cml

Additional supplementary materials:  crystallographic information; 3D view; checkCIF report

## Figures and Tables

**Table 1:** Hydrogen-bond geometry (Å, °)

*D*—H⋯*A*	*D*—H	H⋯*A*	*D*⋯*A*	*D*—H⋯*A*
N1—H1*A*⋯N4	0.86	2.42	3.089 (6)	135
N2—H2*A*⋯N3^i^	0.86	2.21	3.058 (6)	170

Symmetry code: (i) 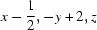.

**Fig. 1.**
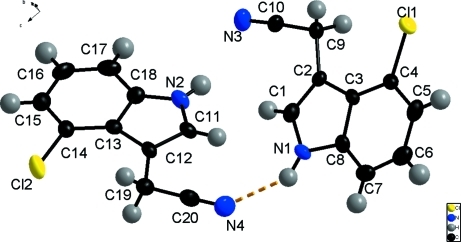
The molecular structure of the title compound showing 30% displacement ellipsoids.

**Fig. 2.**
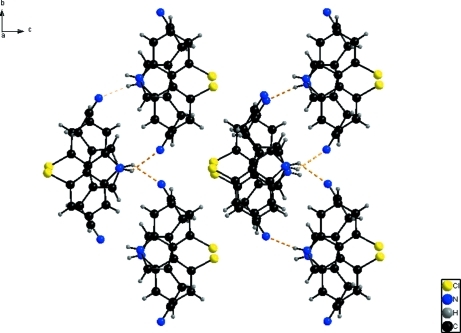
Packing diagram of the title compound, showing the structure from the a axis. Hydrogen bonds are shown as dashed lines.

## supplementary crystallographic information

### Experimental

The title compound 3-(cyanomethyl)indole-4-chlorine was purchased commercially from ChemFuture PharmaTech, Ltd (Nanjing, Jiangsu). and were used as received without further purification. Colourles prisms were obstained by slow evaporation of a methanol solution.

### Refinement

Positional parameters of all H atoms were calculated geometrically and the H atoms were set to ride the C atoms and N atoms to which they are bonded, with *U*ĩso~(H)= 1.2 *U*ĩso~(C, N).C—H atoms were included with bond distances ranging from 0.93 to 0.97 Å. Amide N—H hydrogen atoms were included witha distance set to 0.86 Å.

### Crystal data

**Table:**

C_10_H_7_ClN_2_	*F*(000) = 784
*M**_r_* = 190.63	*D*_x_ = 1.429 Mg m^−^^3^
Orthorhombic, *P**c**a*2_1_	Mo *K*α radiation, λ = 0.71073 Å
Hall symbol: P 2c -2ac	Cell parameters from 4057 reflections
*a* = 7.5091 (15) Å	θ = 2.5–27.5°
*b* = 11.041 (2) Å	µ = 0.38 mm^−^^1^
*c* = 21.380 (4) Å	*T* = 293 K
*V* = 1772.6 (6) Å^3^	Prism, colourless
*Z* = 8	0.33 × 0.25 × 0.20 mm

### Data collection

**Table:**

Rigaku SCXmini diffractometer	2753 reflections with *I* > 2σ(*I*)
Radiation source: fine-focus sealed tube	*R*_int_ = 0.058
graphite	θ_max_ = 27.5°, θ_min_ = 3.3°
CCD_Profile_fitting scans	*h* = −9→9
Absorption correction: multi-scan (*CrystalClear*; Rigaku, 2005)	*k* = −14→14
*T*_min_ = 0.893, *T*_max_ = 0.927	*l* = −27→27
17075 measured reflections	2 standard reflections every 150 reflections
4057 independent reflections	intensity decay: none

### Refinement

**Table:**

Refinement on *F*^2^	Hydrogen site location: inferred from neighbouring sites
Least-squares matrix: full	H-atom parameters constrained
*R*[*F*^2^ > 2σ(*F*^2^)] = 0.048	*w* = 1/[σ^2^(*F*_o_^2^) + (0.0469*P*)^2^ + 0.1573*P*] where *P* = (*F*_o_^2^ + 2*F*_c_^2^)/3
*wR*(*F*^2^) = 0.111	(Δ/σ)_max_ < 0.001
*S* = 1.03	Δρ_max_ = 0.27 e Å^−^^3^
4057 reflections	Δρ_min_ = −0.22 e Å^−^^3^
236 parameters	Extinction correction: *SHELXL97* (Sheldrick, 2008)
1 restraint	Extinction coefficient: 0
Primary atom site location: structure-invariant direct methods	Absolute structure: Flack (1983), 1968 Friedel pairs
Secondary atom site location: difference Fourier map	Flack parameter: 0.66 (10)

### Special details

**Table:**

Geometry. All e.s.d.'s (except the e.s.d. in the dihedral angle between two l.s. planes) are estimated using the full covariance matrix. The cell e.s.d.'s are taken into account individually in the estimation of e.s.d.'s in distances, angles and torsion angles; correlations between e.s.d.'s in cell parameters are only used when they are defined by crystal symmetry. An approximate (isotropic) treatment of cell e.s.d.'s is used for estimating e.s.d.'s involving l.s. planes.
Refinement. Refinement of *F*^2^ against ALL reflections. The weighted *R*-factor *wR* and goodness of fit *S* are based on *F*^2^, conventional *R*-factors *R* are based on *F*, with *F* set to zero for negative *F*^2^. The threshold expression of *F*^2^ > σ(*F*^2^) is used only for calculating *R*-factors(gt) *etc*. and is not relevant to the choice of reflections for refinement. *R*-factors based on *F*^2^ are statistically about twice as large as those based on *F*, and *R*- factors based on ALL data will be even larger.

### Fractional atomic coordinates and isotropic or equivalent isotropic displacement parameters (Å^2^)

**Table:**

	*x*	*y*	*z*	*U*_iso_*/*U*_eq_	
Cl1	0.57185 (18)	0.47362 (9)	−0.10226 (5)	0.0585 (3)	
N1	0.3359 (6)	0.4908 (3)	0.1147 (2)	0.0451 (11)	
H1A	0.3006	0.4743	0.1520	0.054*	
N3	0.4392 (5)	0.9029 (3)	0.05314 (18)	0.0781 (11)	
C1	0.3547 (5)	0.6055 (3)	0.0894 (2)	0.0470 (11)	
H1B	0.3234	0.6773	0.1093	0.056*	
C2	0.4252 (4)	0.5977 (3)	0.0317 (2)	0.0372 (8)	
C3	0.4428 (6)	0.4720 (4)	0.0164 (2)	0.0356 (8)	
C4	0.4979 (4)	0.4026 (3)	−0.03431 (17)	0.0399 (7)	
C5	0.4984 (6)	0.2769 (3)	−0.0313 (2)	0.0528 (12)	
H5A	0.5375	0.2322	−0.0656	0.063*	
C6	0.4413 (6)	0.2172 (4)	0.0222 (3)	0.0571 (13)	
H6A	0.4426	0.1330	0.0233	0.069*	
C7	0.3829 (4)	0.2803 (3)	0.07360 (19)	0.0521 (9)	
H7A	0.3450	0.2411	0.1097	0.062*	
C8	0.3834 (5)	0.4034 (3)	0.0688 (2)	0.0412 (10)	
C9	0.4689 (6)	0.7050 (3)	−0.00949 (18)	0.0454 (10)	
H9A	0.5898	0.6971	−0.0249	0.055*	
H9B	0.3894	0.7056	−0.0452	0.055*	
C10	0.4518 (6)	0.8176 (3)	0.0240 (2)	0.0491 (10)	
Cl2	0.31981 (19)	1.02214 (9)	0.39526 (5)	0.0628 (4)	
N2	0.0887 (6)	1.0076 (3)	0.1779 (2)	0.0540 (13)	
H2A	0.0441	1.0232	0.1417	0.065*	
N4	0.1867 (6)	0.5920 (3)	0.23876 (19)	0.0798 (11)	
C11	0.1204 (5)	0.8969 (4)	0.20034 (19)	0.0454 (10)	
H11A	0.1048	0.8260	0.1775	0.054*	
C12	0.1782 (4)	0.9000 (3)	0.26055 (19)	0.0401 (8)	
C13	0.1928 (6)	1.0261 (4)	0.2749 (2)	0.0385 (9)	
C14	0.2470 (4)	1.0945 (3)	0.32694 (17)	0.0442 (8)	
C15	0.2436 (6)	1.2171 (3)	0.3253 (2)	0.0531 (11)	
H15A	0.2807	1.2608	0.3601	0.064*	
C16	0.1853 (6)	1.2786 (4)	0.2721 (3)	0.0618 (14)	
H16A	0.1840	1.3628	0.2720	0.074*	
C17	0.1308 (4)	1.2175 (4)	0.2208 (2)	0.0594 (10)	
H17A	0.0889	1.2594	0.1861	0.071*	
C18	0.1377 (5)	1.0878 (3)	0.2203 (2)	0.0422 (10)	
C19	0.2208 (6)	0.7932 (3)	0.30169 (19)	0.0468 (10)	
H19A	0.3417	0.8005	0.3172	0.056*	
H19B	0.1410	0.7926	0.3374	0.056*	
C20	0.2024 (7)	0.6794 (4)	0.2670 (3)	0.0579 (12)	

### Atomic displacement parameters (Å^2^)

**Table:**

	*U*^11^	*U*^22^	*U*^33^	*U*^12^	*U*^13^	*U*^23^
Cl1	0.0658 (7)	0.0684 (5)	0.0413 (6)	−0.0070 (6)	0.0135 (6)	−0.0086 (8)
N1	0.050 (3)	0.056 (2)	0.029 (3)	−0.0015 (13)	0.004 (2)	0.0031 (12)
N3	0.124 (3)	0.050 (2)	0.060 (2)	−0.011 (2)	−0.015 (2)	−0.0052 (17)
C1	0.037 (2)	0.043 (2)	0.061 (3)	0.0015 (15)	0.0014 (18)	−0.013 (2)
C2	0.0324 (18)	0.0367 (19)	0.042 (2)	−0.0052 (15)	−0.0034 (17)	−0.0017 (16)
C3	0.030 (2)	0.0480 (19)	0.029 (2)	0.001 (2)	−0.0011 (15)	−0.004 (2)
C4	0.0335 (17)	0.0480 (18)	0.0383 (19)	−0.0033 (16)	0.0004 (14)	−0.0022 (15)
C5	0.050 (2)	0.037 (2)	0.072 (3)	0.0072 (18)	−0.005 (2)	−0.018 (2)
C6	0.050 (2)	0.047 (2)	0.074 (3)	−0.001 (2)	−0.008 (2)	0.005 (2)
C7	0.049 (2)	0.057 (2)	0.051 (2)	−0.0046 (16)	−0.0075 (17)	0.014 (2)
C8	0.042 (2)	0.046 (2)	0.036 (2)	0.0043 (15)	−0.0130 (18)	−0.0005 (16)
C9	0.051 (2)	0.045 (2)	0.040 (2)	−0.0052 (17)	−0.0022 (19)	−0.0067 (16)
C10	0.064 (3)	0.036 (2)	0.047 (2)	−0.0061 (19)	−0.0045 (19)	−0.0002 (19)
Cl2	0.0702 (8)	0.0734 (6)	0.0447 (7)	0.0137 (6)	−0.0164 (6)	−0.0136 (9)
N2	0.056 (3)	0.071 (3)	0.035 (3)	0.0072 (15)	−0.009 (3)	0.0068 (14)
N4	0.122 (3)	0.043 (2)	0.074 (3)	−0.005 (2)	0.009 (2)	−0.0032 (18)
C11	0.050 (2)	0.056 (2)	0.030 (2)	0.0035 (18)	0.0019 (16)	0.0035 (17)
C12	0.0360 (19)	0.054 (2)	0.031 (2)	0.0018 (17)	0.0026 (17)	−0.0042 (17)
C13	0.033 (2)	0.0450 (18)	0.038 (2)	0.003 (2)	0.0077 (16)	0.000 (2)
C14	0.0391 (19)	0.0499 (19)	0.044 (2)	0.0003 (17)	0.0050 (16)	−0.0071 (17)
C15	0.048 (2)	0.053 (2)	0.058 (3)	−0.001 (2)	0.006 (2)	−0.007 (2)
C16	0.066 (3)	0.033 (2)	0.087 (4)	−0.001 (2)	0.022 (3)	0.002 (2)
C17	0.051 (2)	0.061 (2)	0.066 (3)	0.0055 (19)	0.008 (2)	0.023 (2)
C18	0.032 (2)	0.041 (2)	0.053 (3)	0.0096 (15)	0.002 (2)	0.0007 (18)
C19	0.049 (2)	0.045 (2)	0.046 (3)	0.0065 (17)	0.0035 (19)	0.0020 (17)
C20	0.075 (3)	0.052 (2)	0.046 (3)	0.003 (2)	0.009 (2)	0.008 (2)

### Geometric parameters (Å, °)

**Table:**

Cl1—C4	1.742 (4)	Cl2—C14	1.752 (4)
N1—C1	1.385 (5)	N2—C18	1.319 (6)
N1—C8	1.421 (6)	N2—C11	1.335 (5)
N1—H1A	0.8600	N2—H2A	0.8601
N3—C10	1.132 (5)	N4—C20	1.144 (6)
C1—C2	1.346 (5)	C11—C12	1.359 (5)
C1—H1B	0.9300	C11—H11A	0.9300
C2—C3	1.432 (4)	C12—C13	1.430 (5)
C2—C9	1.511 (5)	C12—C19	1.506 (5)
C3—C4	1.391 (5)	C13—C14	1.405 (6)
C3—C8	1.424 (6)	C13—C18	1.413 (6)
C4—C5	1.390 (5)	C14—C15	1.354 (5)
C5—C6	1.388 (7)	C15—C16	1.395 (7)
C5—H5A	0.9300	C15—H15A	0.9300
C6—C7	1.373 (7)	C16—C17	1.351 (7)
C6—H6A	0.9300	C16—H16A	0.9300
C7—C8	1.364 (5)	C17—C18	1.434 (6)
C7—H7A	0.9300	C17—H17A	0.9300
C9—C10	1.441 (5)	C19—C20	1.466 (6)
C9—H9A	0.9700	C19—H19A	0.9700
C9—H9B	0.9700	C19—H19B	0.9700
			
C1—N1—C8	109.0 (4)	C18—N2—C11	108.5 (5)
C1—N1—H1A	125.9	C18—N2—H2A	126.4
C8—N1—H1A	125.1	C11—N2—H2A	125.2
C2—C1—N1	109.9 (4)	N2—C11—C12	112.0 (4)
C2—C1—H1B	125.1	N2—C11—H11A	124.0
N1—C1—H1B	125.1	C12—C11—H11A	124.0
C1—C2—C3	107.9 (3)	C11—C12—C13	104.6 (4)
C1—C2—C9	124.7 (3)	C11—C12—C19	127.0 (3)
C3—C2—C9	127.4 (4)	C13—C12—C19	128.4 (4)
C4—C3—C8	114.4 (3)	C14—C13—C18	118.7 (4)
C4—C3—C2	137.6 (4)	C14—C13—C12	135.7 (4)
C8—C3—C2	107.9 (4)	C18—C13—C12	105.6 (4)
C3—C4—C5	121.0 (3)	C15—C14—C13	120.7 (4)
C3—C4—Cl1	119.8 (3)	C15—C14—Cl2	118.9 (3)
C5—C4—Cl1	119.2 (3)	C13—C14—Cl2	120.4 (3)
C6—C5—C4	120.7 (4)	C14—C15—C16	120.9 (4)
C6—C5—H5A	119.6	C14—C15—H15A	119.5
C4—C5—H5A	119.6	C16—C15—H15A	119.5
C7—C6—C5	121.2 (4)	C17—C16—C15	120.9 (4)
C7—C6—H6A	119.4	C17—C16—H16A	119.5
C5—C6—H6A	119.4	C15—C16—H16A	119.5
C6—C7—C8	116.4 (4)	C16—C17—C18	119.6 (4)
C6—C7—H7A	121.8	C16—C17—H17A	120.2
C8—C7—H7A	121.8	C18—C17—H17A	120.2
C7—C8—C3	126.2 (4)	N2—C18—C13	109.1 (4)
C7—C8—N1	128.6 (4)	N2—C18—C17	131.7 (4)
C3—C8—N1	105.1 (3)	C13—C18—C17	119.0 (4)
C10—C9—C2	111.5 (3)	C20—C19—C12	110.8 (4)
C10—C9—H9A	109.3	C20—C19—H19A	109.5
C2—C9—H9A	109.3	C12—C19—H19A	109.5
C10—C9—H9B	109.3	C20—C19—H19B	109.5
C2—C9—H9B	109.3	C12—C19—H19B	109.5
H9A—C9—H9B	108.0	H19A—C19—H19B	108.1
N3—C10—C9	176.5 (5)	N4—C20—C19	178.4 (5)
			
C8—N1—C1—C2	−4.9 (5)	C18—N2—C11—C12	5.0 (6)
N1—C1—C2—C3	3.9 (5)	N2—C11—C12—C13	−3.6 (5)
N1—C1—C2—C9	−178.4 (4)	N2—C11—C12—C19	177.7 (4)
C1—C2—C3—C4	177.3 (5)	C11—C12—C13—C14	−178.4 (5)
C9—C2—C3—C4	−0.2 (8)	C19—C12—C13—C14	0.3 (8)
C1—C2—C3—C8	−1.5 (4)	C11—C12—C13—C18	0.9 (4)
C9—C2—C3—C8	−179.1 (4)	C19—C12—C13—C18	179.6 (4)
C8—C3—C4—C5	−2.0 (5)	C18—C13—C14—C15	1.2 (6)
C2—C3—C4—C5	179.2 (5)	C12—C13—C14—C15	−179.6 (5)
C8—C3—C4—Cl1	179.3 (3)	C18—C13—C14—Cl2	−179.0 (3)
C2—C3—C4—Cl1	0.5 (7)	C12—C13—C14—Cl2	0.3 (7)
C3—C4—C5—C6	1.0 (6)	C13—C14—C15—C16	0.4 (6)
Cl1—C4—C5—C6	179.7 (4)	Cl2—C14—C15—C16	−179.5 (4)
C4—C5—C6—C7	0.0 (7)	C14—C15—C16—C17	0.0 (7)
C5—C6—C7—C8	0.4 (6)	C15—C16—C17—C18	−1.8 (7)
C6—C7—C8—C3	−1.7 (6)	C11—N2—C18—C13	−4.2 (6)
C6—C7—C8—N1	−177.9 (4)	C11—N2—C18—C17	−179.0 (4)
C4—C3—C8—C7	2.5 (6)	C14—C13—C18—N2	−178.5 (4)
C2—C3—C8—C7	−178.3 (4)	C12—C13—C18—N2	2.0 (5)
C4—C3—C8—N1	179.4 (4)	C14—C13—C18—C17	−3.0 (6)
C2—C3—C8—N1	−1.4 (4)	C12—C13—C18—C17	177.5 (3)
C1—N1—C8—C7	−179.4 (4)	C16—C17—C18—N2	177.7 (5)
C1—N1—C8—C3	3.8 (5)	C16—C17—C18—C13	3.4 (6)
C1—C2—C9—C10	8.9 (6)	C11—C12—C19—C20	3.4 (6)
C3—C2—C9—C10	−173.9 (4)	C13—C12—C19—C20	−174.9 (4)
C2—C9—C10—N3	21 (9)	C12—C19—C20—N4	−3(20)

### Hydrogen-bond geometry (Å, °)

**Table:**

*D*—H···*A*	*D*—H	H···*A*	*D*···*A*	*D*—H···*A*
N1—H1A···N4	0.86	2.42	3.089 (6)	135
N2—H2A···N3^i^	0.86	2.21	3.058 (6)	170

Symmetry codes: (i) *x*−1/2, −*y*+2, *z*.
